# Tudor-domain protein PHF20L1 reads lysine methylated retinoblastoma tumour suppressor protein

**DOI:** 10.1038/cdd.2017.135

**Published:** 2017-08-25

**Authors:** Simon M Carr, Shonagh Munro, Cari A Sagum, Oleg Fedorov, Mark T Bedford, Nicholas B La Thangue

**Affiliations:** 1 Department of Oncology, University of Oxford, Old Road Campus Research Building, Old Road Campus, Roosevelt Drive, Headington, Oxford OX3 7DQ, UK; 2 Department of Molecular Carcinogenesis, The University of Texas, MD Anderson Cancer Center, Smithville, TX 77030, USA; 3 Nuffield Department of Clinical Medicine, Structural Genomics Consortium Oxford, University of Oxford, Old Road Campus Research Building, Old Road Campus, Roosevelt Drive, Headington, Oxford OX3 7DQ, UK

## Abstract

The retinoblastoma tumour suppressor protein (pRb) classically functions to regulate early cell cycle progression where it acts to enforce a number of checkpoints in response to cellular stress and DNA damage. Methylation at lysine (K) 810, which occurs within a critical CDK phosphorylation site and antagonises a CDK-dependent phosphorylation event at the neighbouring S807 residue, acts to hold pRb in the hypo-phosphorylated growth-suppressing state. This is mediated in part by the recruitment of the reader protein 53BP1 to di-methylated K810, which allows pRb activity to be effectively integrated with the DNA damage response. Here, we report the surprising observation that an additional methylation-dependent interaction occurs at K810, but rather than the di-methyl mark, it is selective for the mono-methyl K810 mark. Binding of the mono-methyl PHF20L1 reader to methylated pRb occurs on E2F target genes, where it acts to mediate an additional level of control by recruiting the MOF acetyltransferase complex to E2F target genes. Significantly, we find that the interplay between PHF20L1 and mono-methyl pRb is important for maintaining the integrity of a pRb-dependent G1–S-phase checkpoint. Our results highlight the distinct roles that methyl-lysine readers have in regulating the biological activity of pRb.

pRb is the archetypal tumour suppressor that is directly mutated or its protein product functionally inactivated in the vast majority of human tumours.^1^ It has been ascribed many functions, but one of its primary roles is to regulate transcription of E2F-responsive genes connected with cell cycle progression, DNA replication, and other cell fates including apoptosis and differentiation.^2^ This regulation is mediated by a direct interaction between pRb and the transcriptional activation domain of certain E2F transcription factors, like E2F-1, which hinders transcription and results in growth inhibition.^3, 4^ pRb also mediates active repression by recruiting proteins that modulate chromatin structure, including histone deacetylases, histone methyltransferases and chromatin remodelling factors.^2^

The activity of pRb and its interaction with the E2F family is itself governed by a number of post-translational modifications (PTMs).^5^ In cycling cells, pRb activity is modulated by the activity of cyclin-CDK complexes, which phosphorylate pRb to induce the release of E2F transcription factors. pRb can also undergo additional PTMs, including acetylation and lysine methylation, which further impact on pRb activity.^5, 6, 7, 8^ In particular, the methylation of pRb at residue K810 by the enzyme SET7/9 (SETD7) promotes the hypo-phosphorylated, growth-suppressing state of pRb.^8^ Mechanistically, this occurs by interfering with the association between cyclin-CDK complexes and pRb. CDK phosphorylation occurs on the SPX**K/R** motif, where K810 acts as the essential basic residue in the CDK consensus site centred on S807 (SPL**K**). In addition, methylated K810 is ‘read’ by the tandem tudor domain containing protein 53BP1,^9^ a DNA damage-responsive protein that can also interact with methylated H4K20 and is involved in repairing DNA double-strand breaks (DSBs) via non-homologous end joining (NHEJ).^10^ In the context of its interaction with pRb, 53BP1 integrates the DNA damage response with pRb-mediated cell cycle control.^9^ Indeed, the retinoblastoma family of proteins have also been directly implicated in DNA repair via their interaction with additional NHEJ components such as XRCC5 and XRCC6.^11^

PHD-finger protein 20-like 1 (PHF20L1) is linked with breast and ovarian cancers, where gene amplifications and copy-number aberrations are described.^12, 13, 14^ PHF20L1 protein contains two tudor domains, which have been described to interact with mono-methylated lysine residues in H3K4, H4K20^15^ and DNA methyltransferase-1 (DNMT1).^16^ Furthermore, PHF20L1 is a component of an evolutionarily conserved protein complex containing the human ortholog of the acetyltransferase males absent on the first (MOF).^17^ In human cells, MOF-containing complexes are responsible for histone H4K16 acetylation,^18^ which has been implicated as a key mark in transcriptional regulation.^19, 20, 21, 22^ MOF activity has also been linked with multiple stages of the DNA damage response, as loss of MOF and H4K16 acetylation leads to ionising radiation sensitivity and defective DNA damage repair in mice and human cell lines.^23, 24^

In this report, we elucidate an unexpected level of methylation-dependent control on K810 pRb, in which the mono-methyl mark is read by PHF20L1, contrasting with 53BP1 that reads the di-methyl K810 mark. Significantly, the methylation-dependent recruitment of PHF20L1 to K810me is required for proper recovery of cells from pRb-mediated checkpoint control, enabling them to re-enter the cell cycle. The interaction of PHF20L1 with pRb allows the recruitment of the MOF acetyltransferase complex to E2F target genes. Our results highlight the role of methyl readers in the control of pRb biology and highlight the potential interplay between readers of the methyl mark and acetyltransferases in cell cycle regulation.

## Results

### pRb lysine methylation is read by PHF20L1

**Figure 1:**
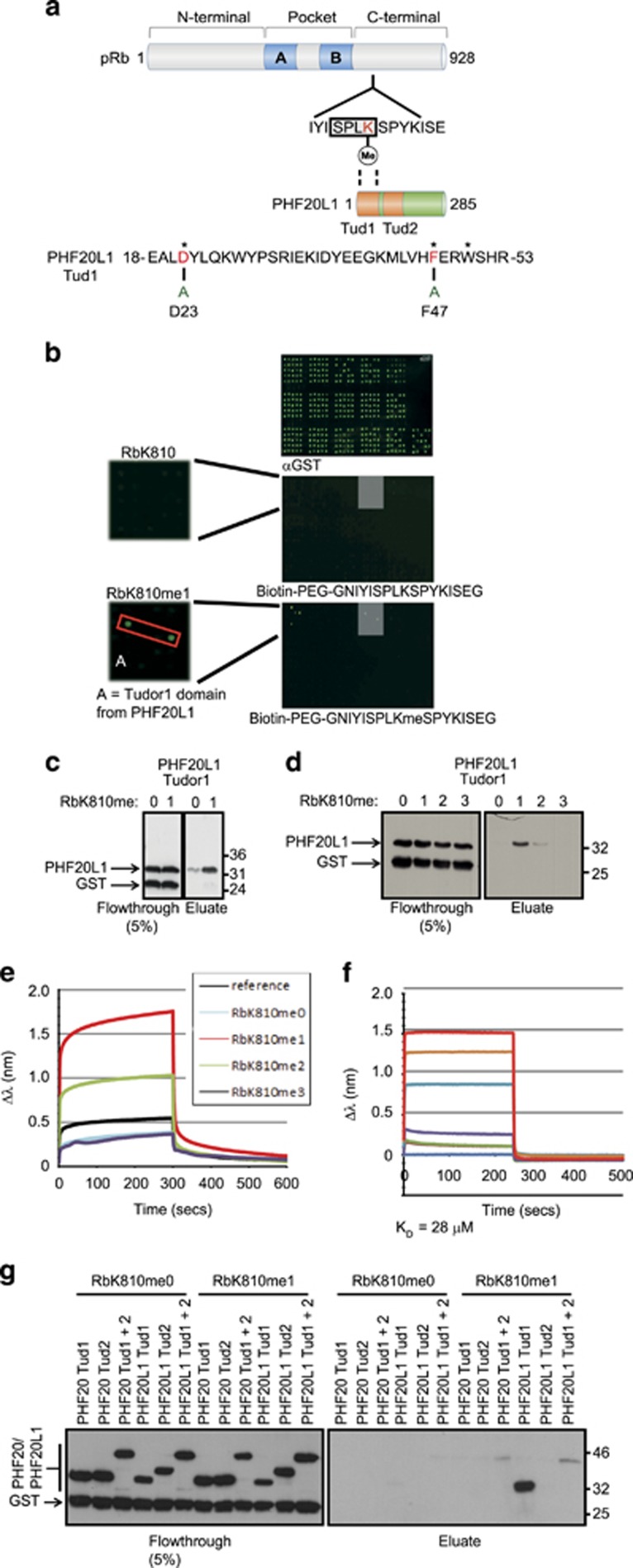
Identification of a new reader protein for pRb methylated at K810. (**a**) Schematic representation of pRb and PHF20L1 proteins. The amino acid sequence around residue K810 (in red) is expanded to indicate the CDK consensus motif SPLK (boxed). A methyl-dependent interaction with the tudor 1 domain of PHF20L1 is indicated. The amino acid sequence of PHF20L1 between residues 18 and 53 is displayed to highlight tudor 1 domain residues important for methyl-lysine recognition (*). The D23 and F47 residues mutated to alanine in this study are also indicated. (**b**) CADOR array probed with anti-GST (top), biotinylated RbK810me0 (middle) or biotinylated RbK810me1 (bottom). The grey boxed regions demarked show binding of the methylated pRb peptide to the tudor 1 domain of PHF20L1. The additional green spots represent the previously described interaction with 53BP1. (**c**) Peptide-binding assay in which RbK810me0 or RbK810me1 peptide was incubated with recombinant GST-PHF20L1 tudor 1. The left hand side displays flow-through from the assay, while the right hand side displays the remaining eluted protein. *n*=3. (**d**) As above, although RbK810me0, -me1, -me2 and -me3 peptides were used. *n*=2. (**e**) Biolayer interferometry real-time kinetic analysis of immobilised RbK810me0, -me1, -me2 and -me3 peptides bound to His-PHF20L1 tudor 1. (**f**) As above, but showing the concentration dependent binding of PHF20L1 tudor 1 with the RbK810me1 peptide. A *K*_D_ value of 28 *μ*M was calculated from these data. (**g**) Peptide-binding assay in which RbK810me0 or RbK810me1 peptides were incubated with recombinant GST-PHF20 (tudor 1, tudor 2 or tudor 1+2) or GST-PHF20L1 (tudor 1, tudor 2 or tudor 1+2). *n*=3

Residue K810 in pRb is a critical residue in controlling pRb-dependent growth control.^8^ We used biotinylated pRb peptides to screen the chromatin-associated domain array (CADOR), a platform developed to identify protein domains that bind modified peptides, which includes tudor, MBT, PHD and chromodomains,^25^ and previously used to identify 53BP1.^9^ When this screen was performed with a mono-methylated K810 pRb peptide, we identified the tudor domain protein PHF20L1 (Figure 1b). Significantly, we confirmed that the interaction between PHF20L1 and pRb was methylation-dependent using an *in vitro* peptide-binding assay, and established that PHF20L1 tudor 1 preferentially bound to a methylated (RbK810me1) peptide (Figure 1c). While PHF20L1 tudor 1 could interact with both mono- and di-methyl K810, it showed a stronger binding efficiency toward the mono-methylated peptide, and failed to bind the tri-methylated K810 peptide (Figure 1d). This observation was confirmed by biolayer interferometry (BLI), which again highlighted a preference for the RbK810me1 peptide (Figure 1e), with a dissociation constant of 28 *μ*M (Figure 1f). Moreover, use of a full-length PHF20L1 protein (containing tudor 1 and tudor 2 domains) showed a similar *K*_D_ of 34 *μ*M, indicating that the second tudor domain of PHF20L1 does not contribute significantly to the interaction with RbK810me1 (Supplementary Figure S1b). Indeed, when the tudor domains of PHF20L1 were expressed individually and used in an *in vitro* peptide-binding assay, only tudor 1 bound to RbK810me1, while the tudor 2 domain did not (Figure 1g). The tudor domains from the closely related PHF20 protein displayed minimal binding to RbK810me1, in both the peptide-binding assay and the CADOR array (Figures 1g and b), demonstrating that the methyl–pRb interaction was specific for a single tudor domain in PHF20L1.

**Figure 2:**
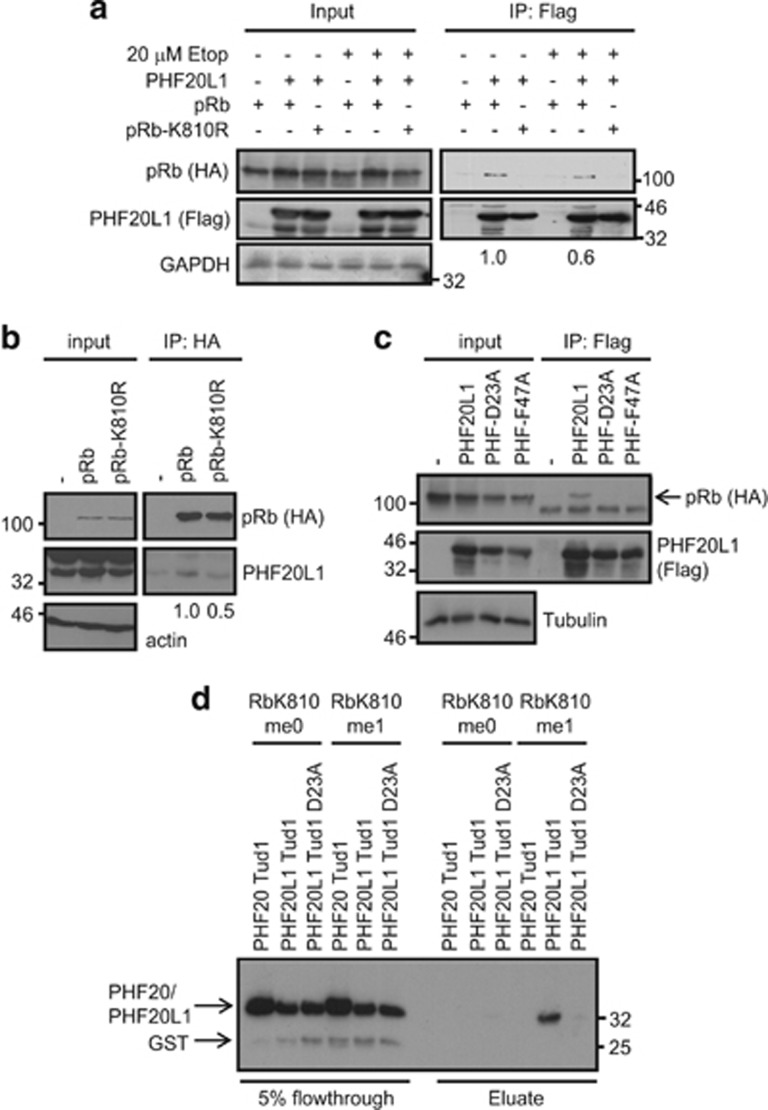
The tudor 1 domain of PHF20L1 shows specificity for RbK810me1. (**a**) SAOS2 cells were transfected with 3 *μ*g of HA-pRb/HA-pRb-K810R and 1 *μ*g of Flag-PHF20L1/empty vector as indicated. Cells were also treated with 20 *μ*M etoposide for 16 h where appropriate. An immunoprecipitation was performed using anti-Flag agarose and co-precipitating pRb was detected by immunoblot. The numbers below the blot indicate the relative amount of pRb co-immunoprecipitated with PHF20L1. *n*=3. (**b**) Extracts from U2OS pRb CRISPR cell lines stably transfected with ectopic empty vector (−), HA-pRb or HA-pRb-K810R were used in an immunoprecipitation with anti-HA agarose. Co-precipitating endogenous PHF20L1 was analysed by immunoblot. The numbers below the blot indicate the relative amount of PHF20L1 co-immunoprecipitated with pRb. *n*=2. (**c**) SAOS2 cells were transfected with 3 *μ*g of HA-pRb and 1 *μ*g of Flag-PHF20L1, Flag-PHF20L1 D23A or Flag-PHF20L1 F47A as indicated. An immunoprecipitation was performed with anti-Flag agarose and co-immunoprecipitating pRb was detected by immunoblot. (**d**) Peptide-binding assay in which RbK810me0 or RbK810me1 peptides were incubated with recombinant GST-PHF20 tudor 1, GST-PHF20L1 tudor 1 or GST-PHF20L1 tudor 1 D23A. The left hand side displays flow-through from the assay, while the right hand side displays the remaining eluted protein. *n*=3

We then expressed pRb and PHF20L1 in SAOS2 (defective for pRb) cells, where an interaction was evident between wild-type pRb, but not the lysine-to-arginine (K810R) mutant, with PHF20L1 (Figure 2a). The interaction was confirmed using a U2OS pRb CRISPR cell line, in which wild-type pRb or the K810R mutant had been reintroduced and expressed ectopically in a stable manner. pRb was observed to co-immunoprecipitate with endogenous PHF20L1, whereas the interaction between PHF20L1 and the K810R mutant was significantly reduced (Figure 2b). Moreover, the interaction required the integrity of the first tudor domain of PHF20L1, as two mutant derivatives (D23A and F47A; Figure 1a) containing substitutions in conserved residues important for methyl-lysine recognition^26^ failed to interact with pRb (Figure 2c). Indeed, in an *in vitro* interaction assay, while wild-type PHF20L1 tudor 1 interacted with an RbK810me1 peptide, the D23A mutant did not (Figure 2d). We also tested the possibility that PHF20L1 recruitment to RbK810me1 was influenced under DNA damage conditions, because previous studies highlighted the DNA damage-dependent reading of di-methyl K810 by 53BP1.^8, 9^ However, cells treated with etoposide displayed a modest reduction in the efficiency on the interaction between PHF20L1 and pRb compared to unperturbed cells (Figure 2a), indicating that PHF20L1 reading of the mono-methyl event is not influenced by DNA damage.

### PHF20L1 recruits the MOF acetyltransferase to mono-methylated pRb

**Figure 3:**
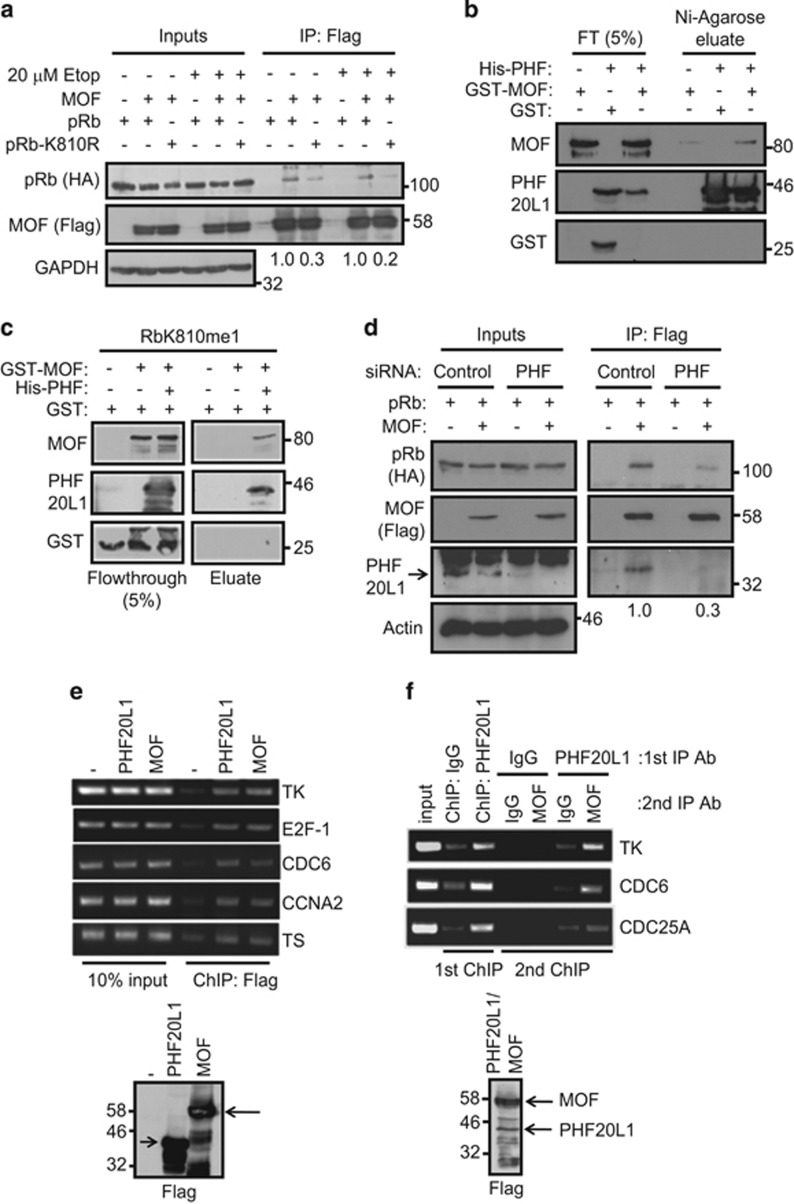
PHF20L1 recruits MOF to methylated pRb. (**a**) U2OS cells were transfected with 3 *μ*g of HA-pRb/HA-pRb-K810R and 1 *μ*g of Flag-MOF/empty vector as indicated. Cells were treated with 20 *μ*M etoposide for 16 h where appropriate. An immunoprecipitation was performed using anti-Flag agarose and co-precipitating pRb was detected by immunoblot. The numbers below the blot indicate the relative amount of pRb co-immunoprecipitated with MOF. *n*=2. (**b**) *In vitro* interaction assay in which 250 ng of His-PHF20L1 was incubated with 250 ng of GST-MOF or GST. His-PHF20L1 was immobilised on Ni-NTA agarose and co-precipitating MOF was detected by immunoblot. *n*=3. (**c**) Peptide-binding assay in which RbK810me1 peptide was incubated with GST-MOF and His-PHF20L1 as indicated. The left hand side displays flow-through from the assay, while the right hand side displays eluted protein. *n*=3. (**d**) U2OS cells were transfected with 20 nM control or PHF20L1 siRNA, followed by 3 *μ*g HA-pRb and 1 *μ*g Flag-MOF/empty vector as indicated. An immunoprecipitation was performed using anti-Flag agarose and co-precipitating pRb was detected by immunoblot. The numbers below the blot indicate the relative amount of pRb co-immunoprecipitated with MOF. *n*=4. (**e**) U20S cells were transfected with 2 *μ*g empty vector (−), Flag-PHF20L1 or Flag-MOF. An immunoprecipitation was performed with anti-Flag antibody and chromatin was analysed by PCR using primers targeting the indicated promoters. *n*=3. (**f**) U2OS cells were transfected with 2 *μ*g of Flag-PHF20L1 and Flag-MOF. Extracts were immunoprecipitated with control IgG or PHF20L1 antibodies (first ChIP). The immunoprecipitated chromatin was then re-immunoprecipitated a second time with control IgG or MOF antibodies, as indicated (second ChIP)

Since PHF20L1 is a component of the MOF acetyltransferase complex in mammalian cells,^17^ we tested the hypothesis that PHF20L1 could recruit MOF to methylated pRb. Initially, we examined whether ectopically expressed pRb and MOF could co-immunoprecipitate in U2OS cells. While MOF was observed to interact with wild-type pRb, the association with the K810R mutant was reduced (Figure 3a), demonstrating that methylation at K810 is important for mediating the pRb-MOF interaction in cells. Once again, DNA damage induced by etoposide treatment did not appear to enhance the efficiency of this interaction (Figure 3a), reciprocating the result observed for the interaction between pRb and PHF20L1 (Figure 2a).

Following on, we tested whether a direct interaction between PHF20L1 and MOF could occur. We performed an *in vitro* interaction assay using recombinant expressed full-length His-tagged PHF20L1 and GST-tagged MOF, where we observed specific binding between the two proteins (Figure 3b). Similarly, in an *in vitro* peptide-binding assay, GST-MOF was only recruited to immobilised RbK810me1 peptide in the presence of His-PHF20L1 (Figure 3c). Finally, to confirm that PHF20L1 was involved in the recruitment of MOF to pRb in cells, we performed an immunoprecipitation experiment under conditions of PHF20L1 depletion through siRNA treatment. The results support the role of PHF20L1 in recruiting MOF to pRb in cells, because while a pRb-MOF interaction was apparent under control siRNA treatment, the interaction was reduced with PHF20L1 siRNA, in both U2OS (Figure 3d) and SAOS2 cells (Supplementary Figure S1c).

### pRb is required for efficient recruitment of PHF20L1-MOF to the promoters of E2F-responsive genes

Given MOF’s role in the regulation of gene expression and chromatin structure, and its de-regulation in a wide variety of cancers,^23^ we reasoned that its interaction with pRb via PHF20L1 would be an important determinant of pRb-dependent growth control. Initially, we identified both ectopically expressed PHF20L1 and MOF by chromatin immunoprecipitation (ChIP) on a number of E2F and pRb-responsive target genes, including thymidine kinase (TK), E2F-1, CDC6, cyclin A2 (CCNA2) and thymidylate synthase (TS) in U2OS cells (Figure 3e). Similar observations were made in the breast cancer cell line MCF-7 (Supplementary Figure S1d). The E2F-responsive promoter association was not regulated by DNA damage, since by ChIP, etoposide-treated cells displayed similar levels of PHF20L1 and MOF as unperturbed cells (Supplementary Figure S1e). Remarkably, in a sequential ChIP analysis, we detected PHF20L1 and MOF together in a chromatin-bound complex on E2F target genes including TK, CDC6 and CDC25A (Figure 3f).

**Figure 4:**
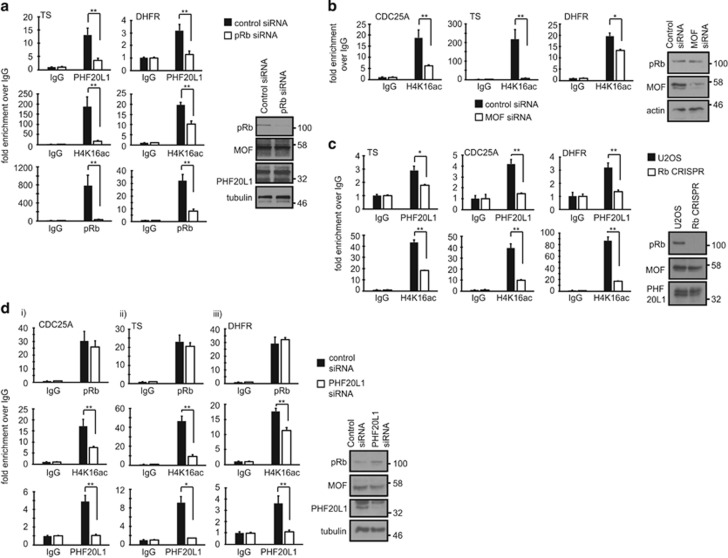
pRb-dependent recruitment of PHF20L1-MOF to the chromatin of E2F-responsive promoters by ChIP analysis. (**a**) U2OS cells were transfected with 20 nM control or pRb siRNA as indicated. Cell extracts were then immunoprecipitated with control IgG, PHF20L1, pRb or H4K16ac antibodies, and chromatin was analysed by qPCR using primers targeting the TS and DHFR promoters. *n*=3 (**b**) U2OS cells were transfected with 20 nM control or MOF siRNA as indicated. Cell extracts were then immunoprecipitated with control IgG or H4K16ac antibodies, and chromatin was analysed by qPCR using primers targeting the indicated promoters. *n*=2. (**c**) U2OS or U2OS pRb CRISPR cells were prepared for ChIP analysis, and immunoprecipitated with control IgG, PHF20L1 or H4K16ac antibodies. Chromatin was analysed by qPCR using primers targeting the indicated promoters. *n*=2. (**d**) U2OS cells were transfected with 20 nM control or PHF20L1 siRNA as indicated. Chromatin immunoprecipitation was then performed using primers against (i) CDC25A, (ii) TS or (iii) DHFR promoters. *n*=3

To determine whether the recruitment of PHF20L1 and MOF to E2F-responsive promoters was dependent upon the presence of pRb, we performed the ChIP analysis in U2OS cells treated with pRb siRNA (Figure 4a). Since we were unable to detect endogenous MOF with the available commercial antibodies by ChIP, we used the presence of a MOF-dependent mark at chromatin as a surrogate for MOF by use of an antibody recognising the H4K16ac mark, as described in previous studies.^27^ We also confirmed that H4K16ac levels reflected the presence of MOF by performing a H4K16ac ChIP under conditions of MOF siRNA treatment, when reduced MOF expression correlated with reduced H4K16ac (Figure 4b). As a control, we tested the effect of pRb siRNA treatment, which caused a reduction in the amount of chromatin-associated pRb on the TS and dihydrofolate reductase (DHFR) promoters (Figure 4a). Most interestingly, this corresponded with a reduction in the promoter association of endogenous PHF20L1 and H4K16ac (Figure 4a). We confirmed this result in a second ChIP experiment, where we compared the chromatin association of PHF20L1 and H4K16ac in wild-type U2OS or U2OS pRb CRISPR cell lines (Figure 4c). Once again, the absence of pRb expression in the CRISPR cell lines correlated with reduced levels of both PHF20L1 and H4K16ac at TS, CDC25A and DHFR promoters, indicating that PHF20L1-MOF recruitment to E2F-responsive promoters is mediated via a pRb-dependent mechanism.

We performed further ChIP experiments in U2OS cells treated with PHF20L1 siRNA, to determine whether PHF20L1 could reciprocally impact on the recruitment of pRb to E2F-responsive promoters (Figure 4d). However, while PHF20L1 siRNA resulted in reduced H4K16ac levels across the promoters tested, consistent with reduced presence of MOF at target genes (Figure 4b), it did not impact on the ability of pRb to localise to these regions of chromatin. Thus, while MOF recruitment to E2F-responsive promoters is strongly dependent on PHF20L1, PHF20L1 is not necessary for pRb to localise to chromatin. PHF20L1-MOF recruitment to TS and DHFR promoters therefore likely occurred because of the ability of PHF20L1 to read methylated pRb (Figure 4a).

### Loss of PHF20L1 impacts cell cycle progression in U2OS cells

**Figure 5:**
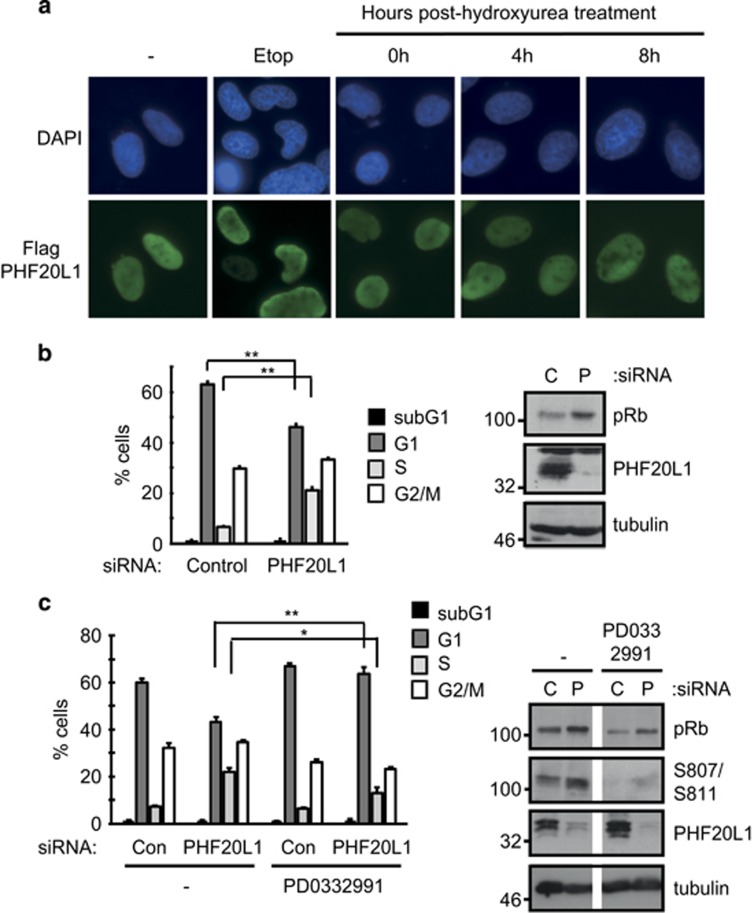
Characterisation of PHF20L1 in cells. (**a**) U2OS cells were seeded on coverslips and transfected with 1 *μ*g of Flag-PHF20L1. Cells were also treated with 20 *μ*M etoposide or 1 mM hydroxyurea for 24 h where indicated. In some cases, cells were released from hydroxyurea block for the indicated time points. Cells were fixed and prepared for immunofluorescence. A flow cytometry analysis of cells is included in Supplementary Figure S2a to demonstrate cell synchronisation. (**b**) U2OS cells were transfected with 20 nM control siRNA (C), or siRNA-targeting PHF20L1 (P). Cells were prepared for flow cytometry analysis. An immunoblot was also performed to monitor input protein levels. *n*=5. (**c**) As above, though cells were treated for 24 h with 2 *μ*M PD0332991 (Cdk4/Cdk6 inhibitor). *n*=3

Given that PHF20L1 remains poorly described, we sought to examine the sub-cellular localisation of the protein throughout different cell cycle stages. Since we were unable to stain for endogenous PHF20L1 protein with the commercially available antibodies, we used FLAG-tagged protein expressed in U2OS cells synchronised at G1–S by treatment with hydroxyurea (Figure 5a).^28^ The cells were then grown in fresh media to permit cell cycle progression, and PHF20L1 localisation was monitored during S and G2/M phases. In all cases, ectopic PHF20L1 was observed to have a nuclear localisation (Figure 5a). The effect of etoposide-induced DNA damage was also examined, though PHF20L1 signal remained nuclear in this context (Figure 5a and Supplementary Figure S2b).

To seek further functional insight, we subsequently examined the effect of PHF20L1 siRNA treatment on the cell cycle profile of U2OS cells (wild-type pRb). Reduced levels of PHF20L1 caused a decline in the observed G1 cell population, with a concomitant increase in the percentage of S- and G2-/M-phase cells (Figure 5b). Interestingly, treatment of cells with PD0332991, a CDK4/CDK6-specific inhibitor that induces pRb hypo-phosphorylation in cells,^29^ abrogated the effect of PHF20L1 siRNA on cell cycle distribution (Figure 5c). These results highlight the possibility that the cellular effect of PHF20L1 is influenced by the phosphorylation status of pRb.

### Cell cycle control by PHF20L1 is mediated in part by a pRb-dependent mechanism

**Figure 6:**
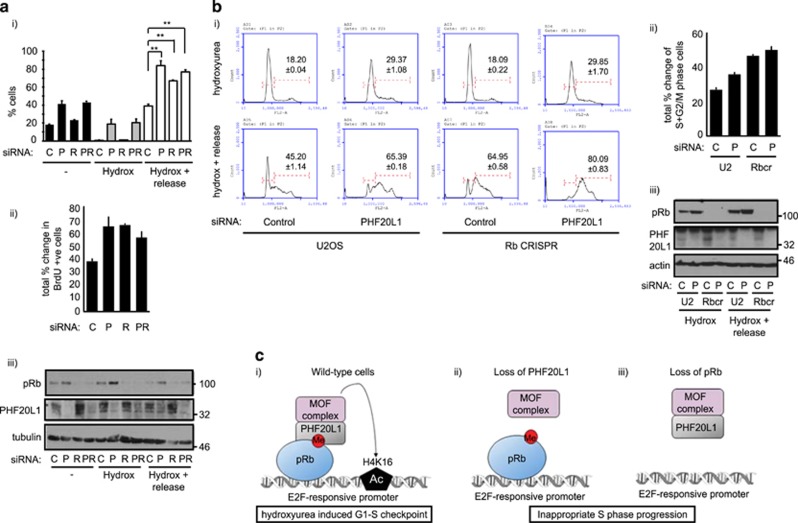
Impact of PHF20L1 and pRb on cell cycle progression. (**a**) (i) U2OS cells were transfected with 20 nM control siRNA (C), or siRNA-targeting PHF20L1 (P), pRb (R) or a combination of both (PR). Some cells were treated 24 h with 1 mM hydroxyurea and released for 2 h. Cells were labelled with BrdU and prepared for flow cytometry analysis. (ii) The total percentage change of BrdU-positive cells between the hydroxyurea treated and released samples was calculated and displayed. (iii) An immunoblot was performed to monitor input protein levels. *n*=2. (**b**) (i) Representative cell cycle profiles taken from a flow cytometry analysis experiment in which U2OS or U2OS pRb CRISPR cell lines (Rbcr) were transfected with 20 nM control siRNA (C) or siRNA-targeting PHF20L1 (P). Cells were treated 42 h with 1 mM hydroxyurea and some released for 8 h. Numbers indicate the mean percentage of cells in S+G2/M phases from the technical repeats within this representative experiment, with S.D. shown. Student’s *t*-tests performed from independent biological replicates indicated that the difference between the U2OS control siRNA sample and the U2OS PHF20L1 siRNA, pRb CRISPR control siRNA, or pRb CRISPR PHF20L1 siRNA samples were all statistically significant (*P*<0.02). (ii) The total percentage change of cells in S+G2/M phases between hydroxyurea treated and released samples was calculated and displayed. (iii) An immunoblot was performed to monitor input protein levels. *n*=3. (**c**) Model for PHF20L1-MOF assembly with methylated pRb on chromatin. In response to pRb mono-methylation, PHF20L1 is recruited to chromatin-bound pRb, where it acts to regulate a pRb-dependent G1–S-phase checkpoint. This checkpoint likely involves the acetyltransferase activity of co-recruited MOF complex, which can target H4K16 at the promoters of E2F-responsive genes (i). In the absence of PHF20L1 (ii) or pRb (iii), this checkpoint response is lost, and cells enter S phase in an inappropriate manner

Next, we decided to further examine whether the cellular effect of PHF20L1 was dependent on the integrity of pRb. To this end, we compared U2OS and its pRb CRISPR cell lines to identify cellular conditions where pRb had an impact on cell cycle regulation. We noticed that pRb CRISPR cell lines displayed a different cell cycle distribution after release from a hydroxyurea treatment as compared to the isogenic U2OS control cells. Specifically, the pRb CRISPR cells showed a higher proportion of S-phase cells after hydroxyurea treatment, whereas wild-type U2OS cells showed many cells remaining in G1 (Supplementary Figure S2c). This was in general agreement with a previous report, where inactivation of pRb enhanced the number of S-phase cells upon release from hydroxyurea.^30^ We therefore used these conditions to perform siRNA-mediated co-depletion studies (Figure 6a) or to examine the impact of PHF20L1 siRNA on the U2OS pRb CRISPR cell lines (Figure 6b).

We monitored the percentage of cells undergoing DNA synthesis by examining the incorporation of BrdU (Figure 6a). While control siRNA-treated cells displayed an increase in BrdU staining from 0.85 to 39.2% upon hydroxyurea release, cells treated with either PHF20L1 or pRb siRNA both demonstrated a much greater increase in BrdU-positive cells (compared to the control treatment) after release from hydroxyurea (+64.9% and +66.05%, respectively) (Figure 6ai and ii). Importantly, an additional effect upon co-depletion of both pRb and PHF20L1 was not apparent (Figure 6a), which supports the hypothesis that pRb and PHF20L1 mediate their effects through a shared pathway.

To further examine the role of pRb and PHF20L1 in cell cycle progression, PHF20L1 levels were reduced in the U2OS and U2OS pRb CRISPR cell lines by siRNA treatment, and cell cycle progression was monitored by flow cytometry (Figure 6b). The pRb CRISPR cell line demonstrated a marked shift to an S+G2-/M-phase population after release from hydroxyurea (+46.86%), as compared to the parental U2OS cells (+27.00%) that still retained a larger number of G1 cells (Figure 6b). Interestingly, a shift to an S+G2-/M-phase population also occurred in U2OS cells treated with PHF20L1 siRNA (+36.02%). When the pRb CRISPR cell line was treated with PHF20L1 siRNA, a large proportion of cells had moved into S or G2/M phases (+50.24%), though it is important to note that the impact of PHF20L1 siRNA was reduced in the pRb CRISPR cells as compared to the parental U2OS. Specifically, the 9.02% increase in S+G2/M-phase cells observed between PHF20L1 and control siRNA-treated U2OS cells was reduced to 3.38% under similar conditions in the pRb CRISPR cell line (Figure 6bii). The fact that the cellular effect of depleting PHF20L1 is reduced in the absence of pRb (Figures 6a and Figures 6b) is consistent with PHF20L1 and pRb acting through over-lapping mechanisms, and compatible with the physical interaction between PHF20L1 and mono-methyl K810 pRb (Figure 2a).

## Discussion

One of the most important functions for the pRb protein in cells is to regulate the transition from G1 into S phase, and this activity is mediated in part by modulating the activity of the E2F transcription factors.^2, 3^ Direct binding of pRb to E2F coincides with an inhibition of transcription and cell cycle arrest, though the recruitment of histone-modifying enzymes by pRb also contributes to this effect.^2^ Recruitment of such complexes often involves the recognition of PTMs, which act as docking sites for proteins containing reader domains.^15^ Growth control by pRb is influenced by different PTMs,^5^ with CDK activity as one principle level of control, driving phosphorylation and inactivation of pRb tumour suppressor activity. Under conditions of cellular stress, CDK-dependent phosphorylation of pRb is inhibited by the induction of CDK inhibitor proteins such as p21, but also via direct methylation of K810 in pRb by the methyltransferase SET7/9.^8^ The recruitment of the tudor domain protein 53BP1 to di-methylated K810 subsequently occurs, enabling pRb activity to be integrated with the DNA damage response.^9^

Here, we have described surprising results on a new level of pRb control, whereby mono-methylated K810 acts to recruit the PHF20L1 methyl reader to E2F-responsive promoters, an interaction that we have found is important for S-phase control (Figure 6c). It is interesting that, in contrast to 53BP1 which is specific for di-methylated K810,^9^ PHF20L1 preferentially binds to the mono-methylated form (Figures 1 and 2). This indicates the degree of K810 methylation dictates reader protein recruitment, permitting pRb growth control to be modulated and fine-tuned in response to discrete stimuli. Indeed, 53BP1 is recruited to pRb in response to DNA damage,^9^ while the recruitment of PHF20L1 was observed to occur in unperturbed cells (Figure 2a).

Interestingly, an analogous situation has been described for the p53 tumour suppressor, which similarly can be mono-methylated at K382 in unperturbed cells, yet di-methylated at the same site in response to DNA damage.^31, 32^ p53K382me1 is involved in transcriptional repression via the recruitment of the MBT-domain-containing protein L3MBTL1,^31^ yet p53K382me2 stabilises p53 levels during the DNA damage response and recruits 53BP1.^32^ SET7/9 is the enzyme responsible for generating RbK810me1 in cells,^8^ though it is possible that other methyltransferases can convert this mark to the di-methylated form, analogous to the H4K20 methylation event. In cells, H4K20me1 is mediated by KMT5A, while the enzymes MMSET/WHSC1, KMT5B and KMT5C can convert the H4K20 mark to higher-order states of methylation.^33, 34, 35^

Since PHF20L1 has been identified as part of the MOF acetyltransferase complex,^17^ we also examined whether pRb and MOF could functionally interact (Figure 3). We found a K810-dependent association between pRb and MOF in cells, and ascertained that the recruitment of MOF to methylated pRb required the presence of PHF20L1 (Figures 3c and d). MOF is intimately linked with transcriptional regulation in mammalian cells,^19, 20, 21, 22, 23^ where it is involved in the activation of genes involved in autophagy and cell cycle progression.^19, 20^ However, it is important to note that ablation of MOF in cells has been linked with both the upregulation and downregulation of gene expression,^21, 22^ suggesting that MOF can act as a negative regulator in some contexts. Indeed, H4K16ac has been shown to recruit the NoRC chromatin remodelling complex to silence a fraction of mammalian rRNA genes, via the establishment of heterochromatin.^36, 37^ Since pRb is also known to associate with chromatin remodelling factors and methyltransferases involved in establishing heterochromatin,^38, 39^ the recruitment of PHF20L1-MOF to E2F-responsive genes may also have an important role in this aspect of pRb-mediated transcriptional control.

The PHF20L1/MOF interaction with pRb appeared to be particularly important during the cellular response to hydroxyurea, a condition under which pRb was observed to impact on cell cycle progression (Figure 6).^30^ Loss of PHF20L1 or pRb caused a higher proportion of S-phase cells after hydroxyurea treatment, while co-depletion of pRb and PHF20L1 did not cause an additive effect on cell cycle distribution. This supports the hypothesis that pRb and PHF20L1/MOF mediate their effects through a shared pathway to regulate appropriate S-phase control (Figure 6c). However, we cannot exclude the possibility that PHF20L1 loss might also influence the stability of the K810 methyl mark, since loss of the reader protein could lead to further methylation/demethylation of K810. Since pRb methylation promotes the hypo-phosphorylated form of pRb,^8^ loss of PHF20L1 could in part influence cell cycle distribution via changes to pRb phosphorylation status, in addition to reduced recruitment of MOF to E2F target genes.

In conclusion, our study describes for the first time the interplay between mono-methylated K810 of pRb and its reader protein PHF20L1. Our results establish the role of mono-methyl K810 to be connected with pRb-dependent S-phase checkpoint control in response to hydroxyurea. Significantly, PHF20L1 recruits the MOF acetyltransferase complex, in turn highlighting an unexpected interplay between MOF, PHF20L1 and pRb growth control. Our results thus indicate the important role that both the site and type of lysine methylation event can have on dictating the biological properties of pRb.

## Materials and methods

### Plasmids and expression vectors

pSG5-HA-pRb and pSG5-HA-pRb-K810R have been described previously.^8^ p3xFlag-CMV-PHF20L1 was generated by cloning PHF20L1 transcript variant 3 from cDNA synthesised from U2OS cells using oligo-d(T)_16_ primer and Superscript III reverse transcriptase (Thermo Fisher; Waltham, MA, USA). Primers corresponding to the ATG start and TGA stop of PHF20L1 were used in the subsequent PCR reaction, and the resulting product was gel purified and ligated into p3xFLAG-CMV-7.1 vector (Sigma; St. Louis, MI, USA). Mammalian and bacterial expression vectors for FLAG-MOF and GST-MOF were kindly donated by Y. Dou (University of Michigan, USA). pGEX-PHF20L1 tudor 1 (1–74), pGEX-PHF20L1 tudor 2 (54–160), pGEX-PHF20L1 tudor 1+2 (1–150), pGEX-PHF20 tudor 1 (1–83), pGEX-PHF20 tudor 2 (58–148) and pGEX-PHF20 tudor 1+2 (1–148) were donated by MTB. pET28a-PHF20L1 tudor 1 and pNIC-Bsa4-PHF20L1 (full length) were generated by subcloning the relevant sequence of PHF20L1 into a pET28a (Novagen, Merck; Darmstadt, Germany) or pNIC-Bsa4 vector (donated by OF). pGEX-PHF20L1 tudor 1 D23A, p3xFlag-CMV-PHF20L1 D23A and p3xFLAG-CMV-PHF20L1 F47A plasmids were all generated with the use of a site-directed mutagenesis kit (Stratagene; San Diego, CA, USA).

### Tissue culture and transfections

U2OS (ATCC no. HTB-96), SAOS2 (ATCC no. HTB-85) and MCF-7 (ATCC no. HTB-22) cells (ATCC; Manassas, VA, USA) were cultured in Dulbecco’s modified Eagle medium (Sigma), supplemented with 10% (v/v) FBS and penicillin/streptomycin. Transfections were performed for 72 h using Genejuice (Novagen, Merck) according to the manufacturer’s instructions. RNA interference was performed using 20 nM of PHF20L1 (s27443), pRb (a combination of two siRNA sequences: siRbA – 5′-UGGUUCACCUCGAACACCC-3′, siRbB – 5′-UUCCUCCACACACUCCAGU-3′), MOF (5′-UGCUGUACAGAAGAACUCA-3′) (all Ambion, Thermo Fisher, Waltham, MA, USA) or GFP siRNA (Dharmacon; Lafayette, CO, USA) for 96 h in oligofectamine (Invitrogen, Thermo Fisher, Waltham, MA, USA) as per the manufacturer’s instructions. U2OS pRb CRISPR cell lines were generated using the method described by Ran *et al.*^40^ U2OS pRb CRISPR cell lines stably expressing ectopic pRb or pRb-K810R were generated by transfecting the CRISPR cells with 2 *μ*g pSG5-HA-pRb/pSG5-HA-pRb-K810R and 0.2 *μ*g pcDNA3.1 (Thermo Fisher) to permit selection in 150 *μ*g/ml G418 (Santa Cruz; Dallas, TX, USA). All cell line stocks were tested for mycoplasma contamination after their generation and prior to liquid nitrogen storage.

### Immunoblotting and immunoprecipitation

The following antibodies were used in immunoblots: anti-pRb (4H1), anti-phospho-pRb S807/S811 (D20B12) (both Cell Signalling; Danvers, MA, USA), anti-GST (B-14), anti-GAPDH (V-18), anti-α-tubulin (TU-02), anti-His probe (H-15) (all Santa Cruz), anti-actin, anti-FLAG M2 (both Sigma), anti-HA (Covance; Princeton, NJ, USA), anti-MOF (A300-992A) (Bethyl Laboratories; Montgomery, TX, USA) and anti-PHF20L1 (HPA028417) (Sigma). For immunoprecipitation, cell extracts were prepared in modified RIPA buffer (50 mM Tris pH 7.5, 150 mM NaCl, 1 mM EDTA, 1% Igepal CA-630 (v/v), 1 mM NaF, 1 mM Na_3_VO_4_, 1 mM AEBSF, protease inhibitor mixture) and incubated with anti-Flag M2 affinity gel or HA agarose (both Sigma) for 2 h at 4 °C. The resin was washed using modified RIPA and eluted with 2 × SDS-loading buffer.

### Protein expression

Plasmids were transformed into BL21 (DE3) bacterial cells and colonies were cultured in terrific broth (Sigma) containing appropriate antibiotics. Protein expression was induced by the addition of 1 mM IPTG for 20 h at 20 °C. Bacteria were then collected and GST- or His-tagged proteins were purified as described previously.^9^

### CADOR array screening

The generation of protein microarrays has been described,^15^ and a list of the protein domains on the array has been published.^25^ Peptides were synthesised as biotin-PEG-GNIYISPLKSPYKISEG and biotin-GNIYISPLK[me]SPYKISEG. Biotinylated peptides were labelled as described.^15^

### Biolayer interferometry

BLI was performed as described,^9^ with the following changes: His-PHF20L1 tudor 1 and full-length His-PHF20L1 samples were prepared in seven 2.5-fold dilutions starting from 200 *μ*M, and measurements were performed using a 250 s association step followed by a 250 s dissociation step on a black 384-well plate.

### Peptide pull-down assay

Peptides were synthesised and immobilised on streptavidin-agarose resin (Thermo Fisher) as described previously.^9^ 250 ng of recombinant GST and 250 ng of GST-PHF20L1 (tudor 1, tudor 2, or tudor 1+2) or GST-PHF20 (tudor 1, tudor 2, or tudor 1+2) was incubated with the immobilised peptides for 30 min at 4 °C. Flow-though was removed, and the resin was washed eight times with modified RIPA buffer containing 300 mM NaCl. Bound protein was eluted using 80 *μ*l of 2 × SDS-loading buffer.

### *In vitro*-binding assays

250 ng of recombinant GST-MOF and 250 ng of His-PHF20L1 were incubated with 15 *μ*l of Ni-NTA resin (Qiagen; Hilden, Germany) in 500 *μ*l of modified RIPA buffer (containing 300 mM NaCl) for 30 min at 4 °C. Flow-through was removed, and the resin was then washed eight times in modified RIPA (300 mM NaCl). Bound protein was eluted using 80 *μ*l of 2x SDS-loading buffer.

### Chromatin immunoprecipitation

Cells were collected and resuspended in PBS containing 1.5 mM ethylene glycol bis(succinimidyl succinate) (EGS; Sigma) for 30 min at room temperature. Following EGS crosslinking, formaldehyde (1% v/v) was added for a further 15 min, prior to neutralisation with glycine (0.125 M). Cells were washed in PBS and lysed first in buffer I (10 mM Tris pH 8, 200 mM NaCl, 1 mM EDTA, 0.5 mM EGTA, protease inhibitor mixture) for 10 min, followed by 10 min in buffer II (10 mM Tris pH 8, 100 mM NaCl, 1 mM EDTA, 0.5 M EGTA, 0.1% Na-deoxycholate (w/v), 0.5% Na-lauroylsarcosine (v/v), protease inhibitor mixture). Chromatin was then sonicated prior to the addition of 1% Igepal CA-630 (v/v). ChIPs were performed using 3 *μ*g of appropriate antibody (control IgG, anti-pRb [4H1], anti-PHF20L1 [HPA028417], anti-H4K16ac [39167] (Active Motif; Carlsbad, CA, USA)) and pre-blocked protein A beads. Two washes with low-salt buffer (20 mM Tris pH 8, 150 mM NaCl, 2 mM EDTA, 0.1% SDS (w/v), 1% Triton X-100 (v/v)), four washes with lithium chloride buffer (10 mM Tris pH 8, 250 mM LiCl, 1 mM EDTA, 1% Igepal CA-630 (v/v), 1% Na-deoxycholate (w/v)) and two washes with TE buffer were performed prior to elution in 2 × 250 *μ*l 1% SDS (w/v), 0.1 M NaHCO_3_ at 65 °C for 15 min. A final concentration of 200 mM NaCl, 10 mM EDTA and 40 mM Tris pH 6.5 was added to each eluate along with RNAse A (20 *μ*g/ml) for 3 h at 55 °C. Crosslinks were then reversed overnight at 65 °C before a 3 h proteinase K (200 *μ*g/ml) treatment at 55 °C. DNA was purified and real-time PCR was performed with Brilliant III Ultra-Fast SYBR in an MX300P QPCR instrument (Agilent; Santa Clara, CA, USA). DNA occupancy was investigated on the E2F-responsive TS, DHFR, CYCA and CDC25A gene promoters from triplicate samples. 5% of the total chromatin fraction used in the immunoprecipitation was used to standardise the ChIP signals observed (ChIP/input) and the results were expressed as fold enrichment over IgG control. In all cases, the presented figure represents results combined from independent biological repeat experiments (*n* as indicated in the figure legends) and displays mean enrichment with S.E. Semi-quantitative PCR was performed using Paq5000 DNA polymerase (Agilent) and agarose gel electrophoresis with primers targeting the indicated promoters.

### Cell cycle analysis

Cells were collected with trypsin and fixed in 70% ethanol in PBS overnight at 4 °C. Fixed cells were resuspended in 40 *μ*g/ml propidium iodide (Sigma) containing 200 *μ*g/ml RNAse A (Sigma) for 1 h. Cell cycle analysis was performed by measuring fluorescence in the FL2 channel using an Accuri C6 flow cytometer and C6 flow software package (Becton Dickinson; Franklin Lakes, NJ, USA). A minimum of 2 × 10^4^ events were collected from duplicate samples. Unless otherwise stated, the presented figure represents results combined from independent biological repeat experiments (*n* as indicated in the figure legends) and displays mean percentage of cells in each cell cycle phase, with S.E. shown.

### BrdU staining

U2OS cells were transfected with the indicated siRNAs and treated with 1 mM hydroxyurea for the last 24 h where indicated. In treatments where release from hydroxyurea-induced arrest was required, cells were washed four times in PBS and fresh media was applied for 2 h. Bromodeoxyuridine (BrdU) of 10 *μ*M (Becton Dickinson) was added to the media for 1 h prior to trypsinisation and fixation in 70% ethanol in PBS overnight at 4 °C. Cells were washed and treated for 30 min with 2 N HCl, then with 0.1 M sodium tetraborate for 10 min. Cells were blocked for 30 min in blocking buffer (1% bovine serum albumin (w/v), 0.1% Triton X-100 (v/v), in PBS), then labelled with 20 *μ*l anti-BrdU-FITC antibody (BD Pharmingen) in 100 *μ*l blocking buffer for 30 min. Three washes were performed before resuspending cells in propidium iodide/RNAse A buffer as described for cell cycle analysis. A minimum of 2 × 10^4^ events were collected from duplicate samples. The displayed figure is a representative experiment with results expressed as average percentage of BrdU-positive cells, with S.E. shown. The *n* value quoted in the figure legend represents the number of independent biological repeat experiments performed.

### Fluorescence microscopy

Cells grown on 13 mm coverslips were fixed in 4% paraformaldehyde (w/v) in PBS for 15 min, prior to permeabilisation in 0.5% Triton X-100 (v/v) in PBS for 15 min. Cells were then blocked in blocking buffer for 1 h, prior to 1 h labelling with primary antibody at 1 : 500 dilution (anti-HA (Y-11) (Santa Cruz) or anti-Flag antibody). Cells were washed three times before being stained with secondary antibody for 1 h (anti-mouse and anti-rabbit Alexa Fluor 488 and 594 antibodies (Thermo Fisher)). Cells were washed three more times and then mounted on glass slides using Vectorshield mounting media containing DAPI (Vectorlabs; Peterborough, UK). Images were collected using Openlab5 software (Improvision; Coventry, UK) and an Olympus BX60 fluorescent microscope fitted with a Hamamatsu C4742-95 camera.

### Statistical analyses

Statistical analyses was performed using a two-tailed, unpaired Student’s *t*-test with Excel software (Microsoft; Redmond, WA, USA). Unless otherwise indicated, data are shown as means with S.E. displayed. *P*-values are indicated as **P<0.02 or *P<0.05.
