# Supplementary figures and images for: Tudor-domain protein PHF20L1 reads lysine methylated retinoblastoma tumour suppressor protein
Source: Cell Death Differ. 2017 Aug 25;24(12):2139–49. doi: 10.1038/cdd.2017.135 (PMC5686351; doi:10.1038/cdd.2017.135)

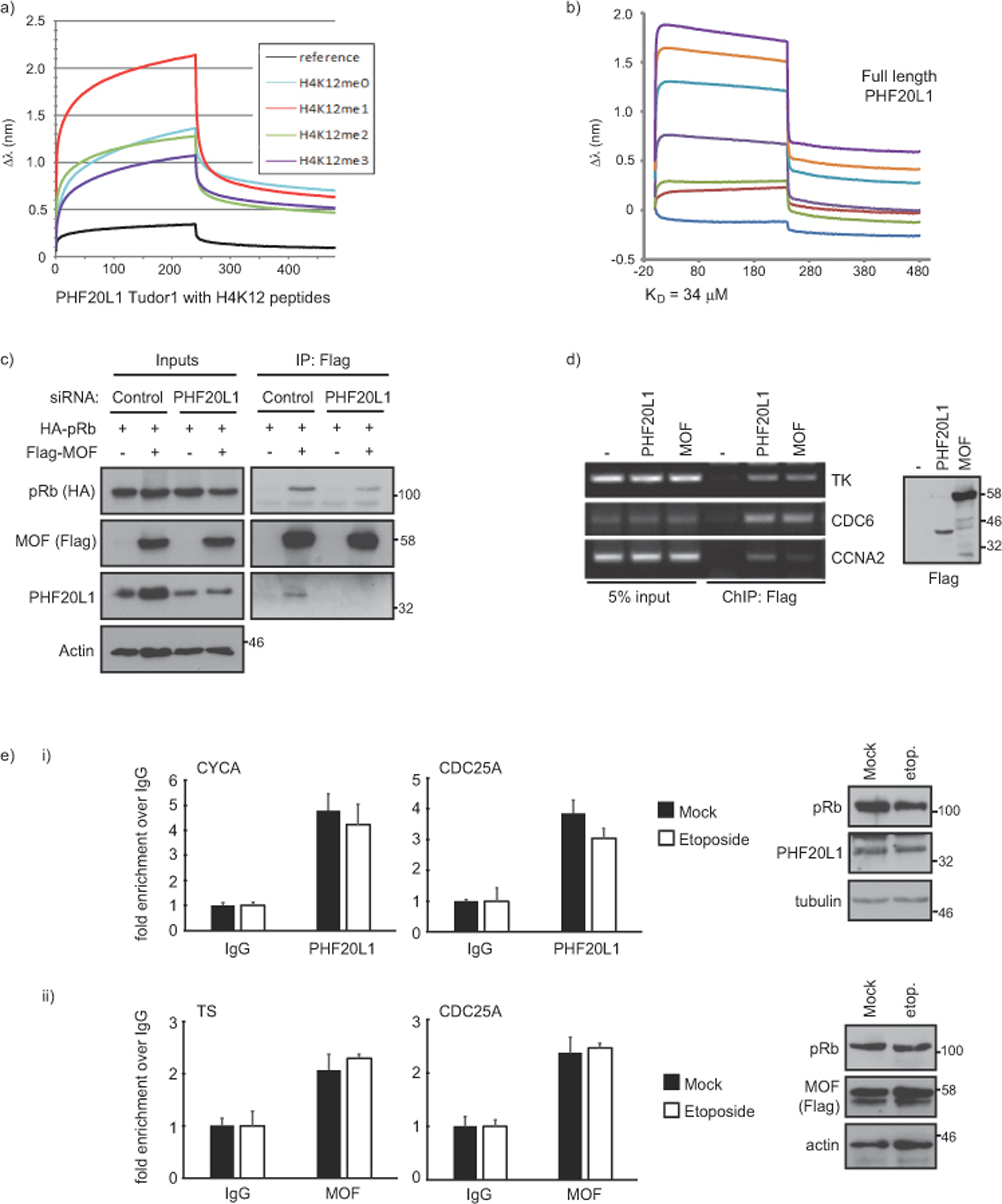

Supplement: Supplementary Figure 1 [file cdd2017135x1.tif]

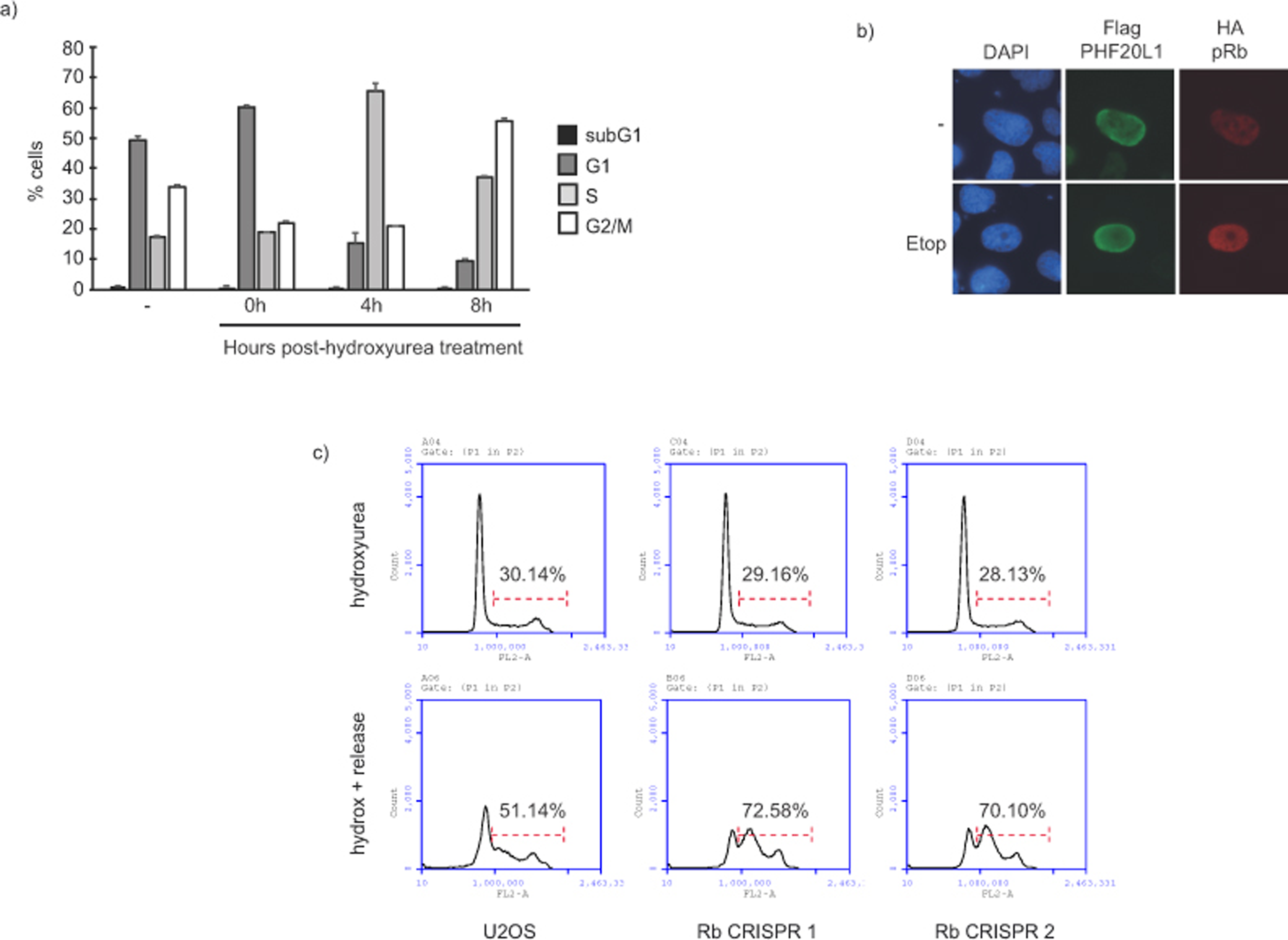

Supplement: Supplementary Figure 2 [file cdd2017135x2.tif]
